# Supplementary material for: A pyrolysis-free Ni/Fe bimetallic electrocatalyst for overall water splitting
Source: Nat Commun. 2023 Mar 31;14:1792. doi: 10.1038/s41467-023-37530-9 (PMC10063682; doi:10.1038/s41467-023-37530-9)
Supplement: Supplementary file 1 — Supporting Information [file 41467_2023_37530_MOESM1_ESM.pdf]

Supporting Information

A pyrolysis-free Ni/Fe bimetallic electrocatalyst for overall water splitting

Ying Zang,[1,2] Di-Qiu Lu,[1] Kun Wang,[2,3] Bo Li,[3] Peng Peng,[2] Ya-Qian Lan,[1] Shuang-Quan Zang[2*]*

*Corresponding author. E-mail: 13020010407@163.com, zangsqzg@zzu.edu.cn

Supplementary Figures

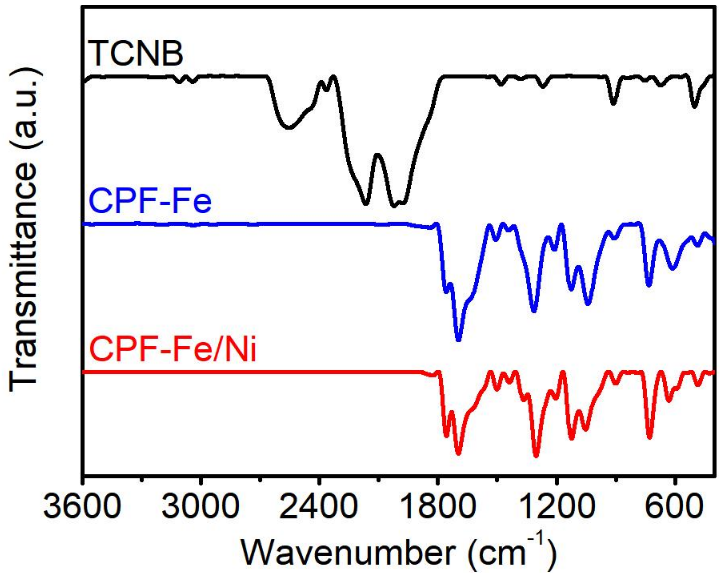

Supplementary Fig. 1. FT-IR of TCNB, CPF-Fe and CPF-Fe/Ni.

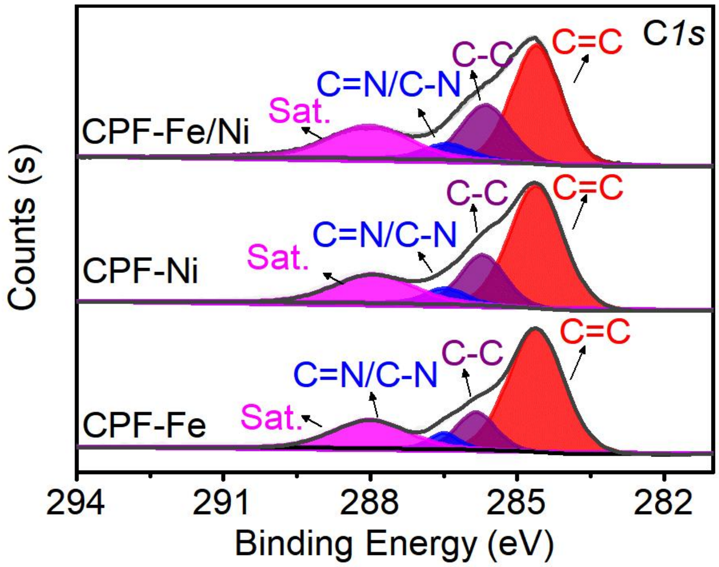

Supplementary Fig. 2. High-resolution C1s spectra of CPF-Fe and CPF-Fe/Ni.

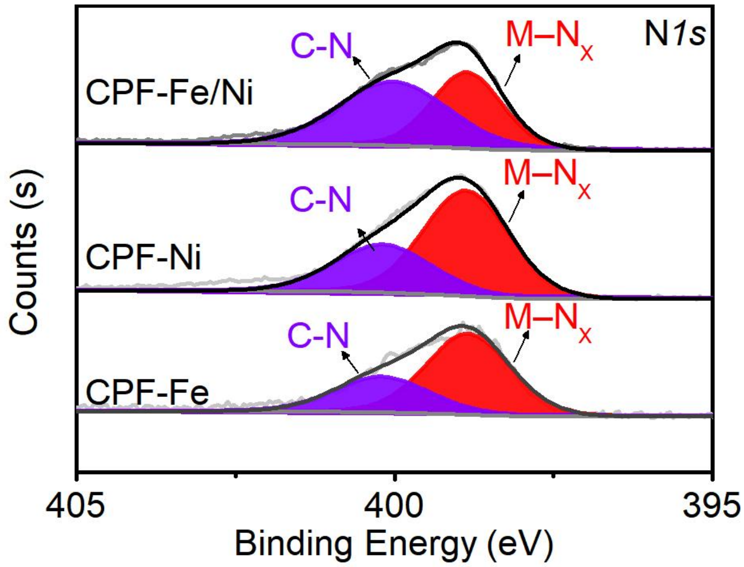

Supplementary Fig. 3. High-resolution N1s spectra of CPF-Fe and CPF-Fe/Ni.

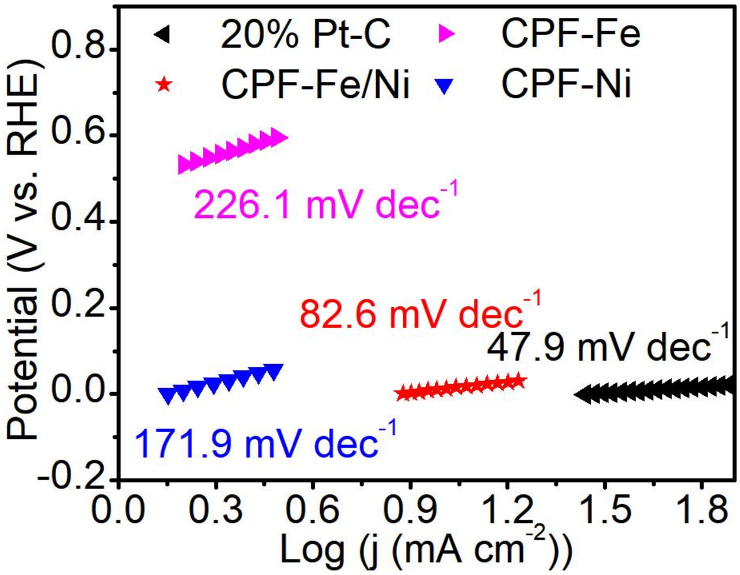

Supplementary Fig. 4. Tafel slopes for 20% Pt-C, CPF-Fe, CPF-Ni and CPF-Fe/Ni as HER catalysts in 0.5 M H_2SO_4.

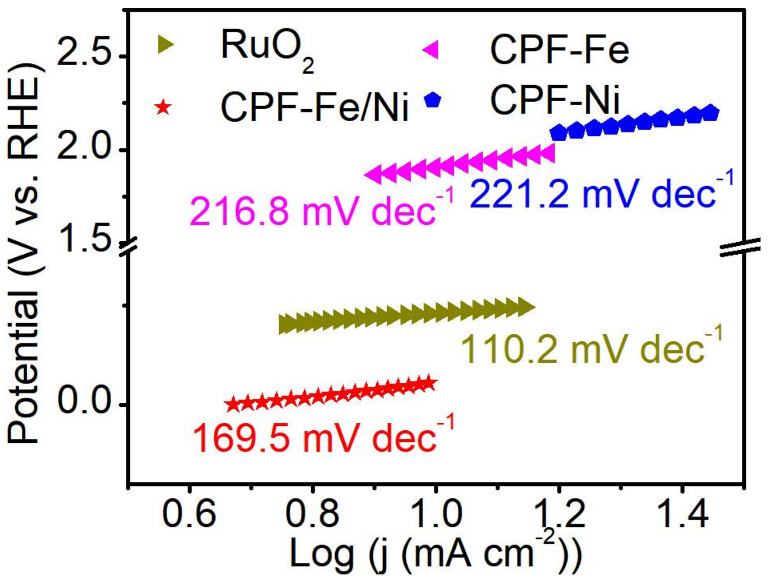

Supplementary Fig. 5. Tafel slopes for RuO_2, CPF-Fe, CPF-Ni and CPF-Fe/Ni as OER catalyst in 0.5 M H_2SO_4.

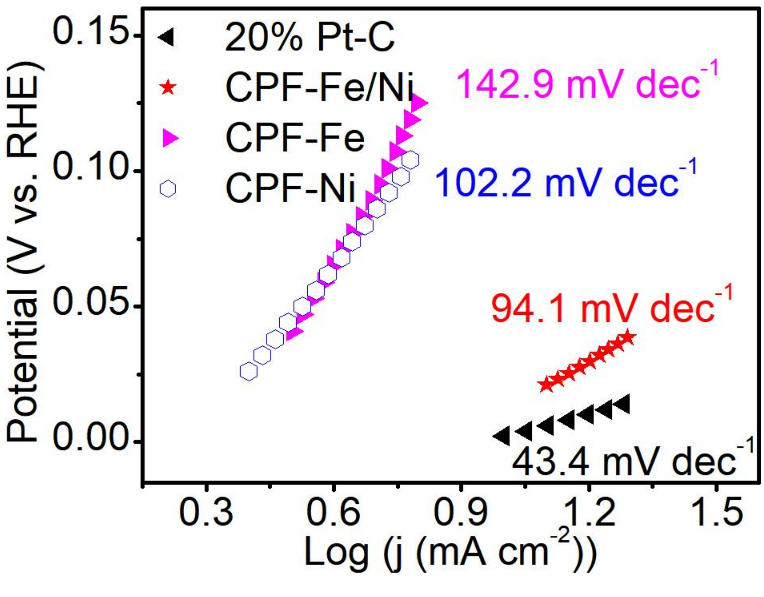

Supplementary Fig. 6. Tafel slopes for 20% Pt-C, CPF-Fe, CPF-Ni and CPF-Fe/Ni as HER catalyst in 1 M KOH.

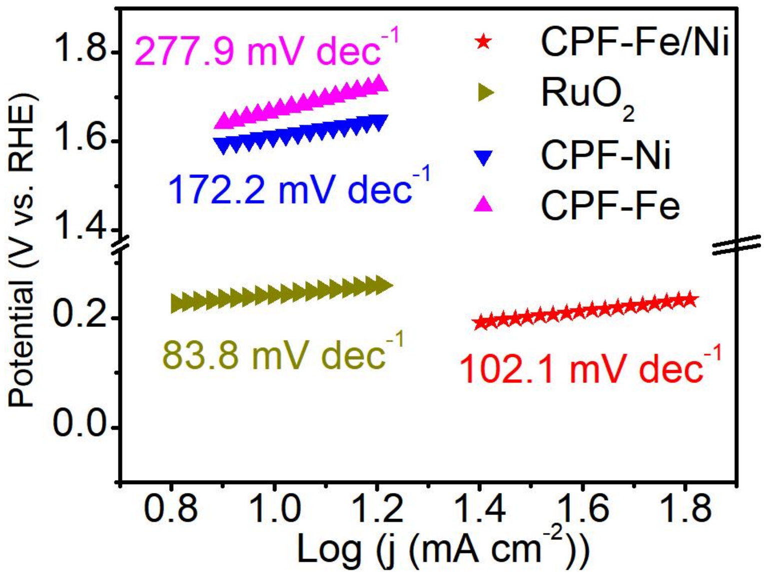

Supplementary Fig. 7. Tafel slopes for RuO_2, CPF-Fe, CPF-Ni and CPF-Fe/Ni as OER catalyst in 1 M KOH.

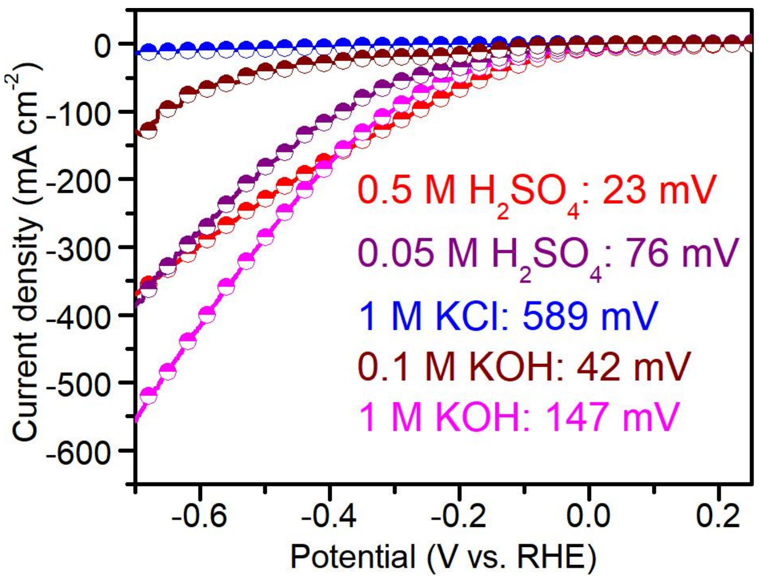

Supplementary Fig. 8. HER polarization curves with a speed of 10 mV s^{-1} in a wide pH.

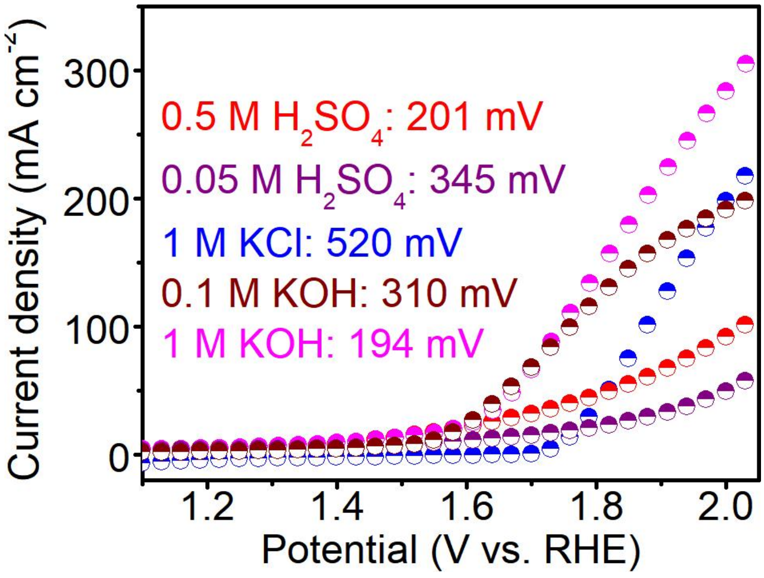

Supplementary Fig. 9. OER polarization curve with a speed of 10 mV s^{-1} in a wide pH.

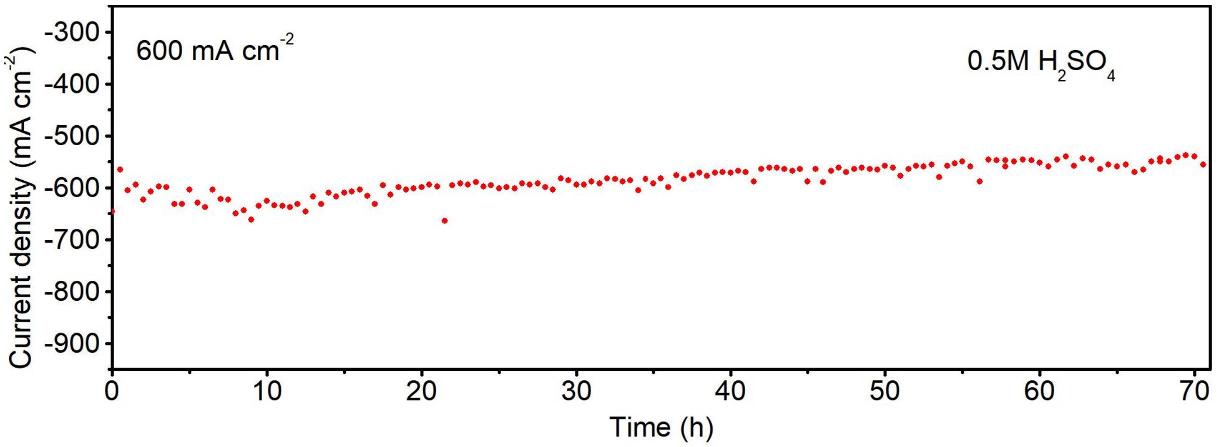

Supplementary Fig. 10. The chronopotentiometry of CPF-Fe/Ni in 0.5 M H_2SO_4 at 600 mA cm^{-2}.

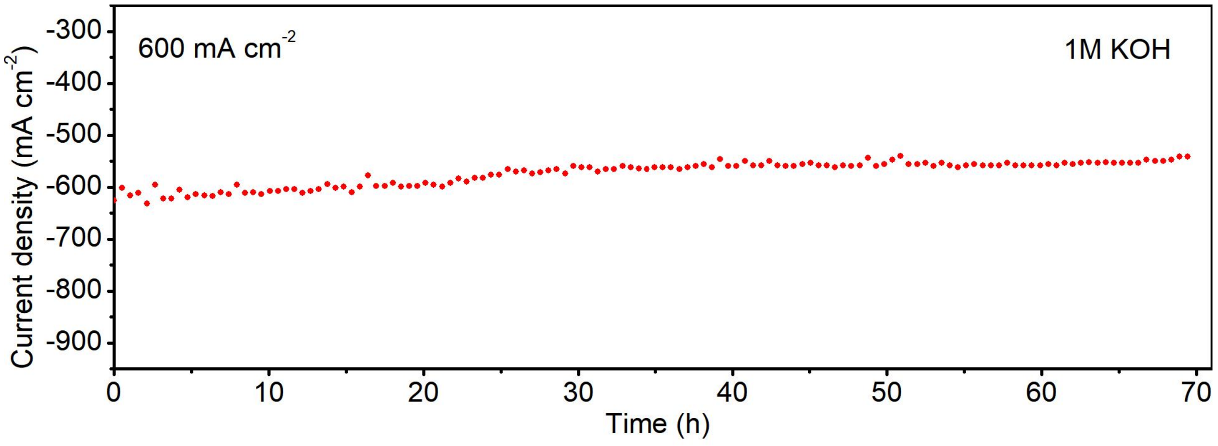

Supplementary Fig. 11. The chronopotentiometry of CPF-Fe/Ni in 1 M KOH at 600 mA cm^{-2}.

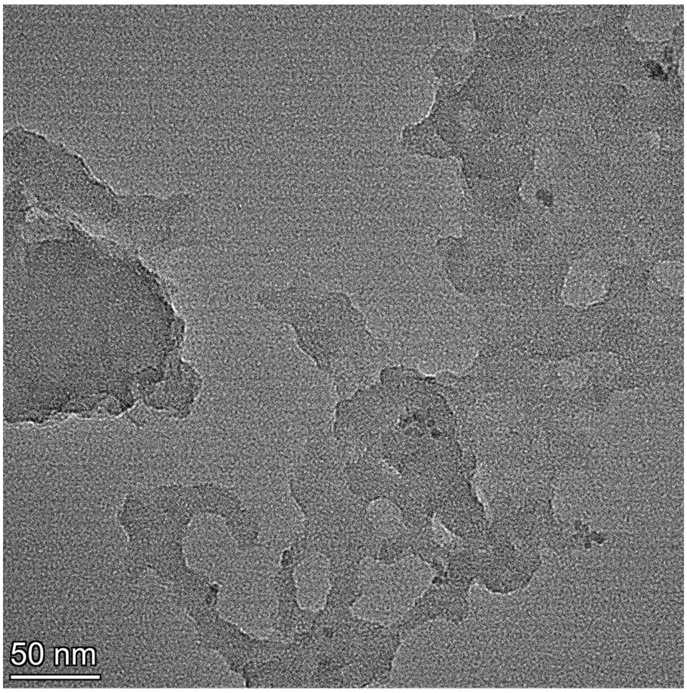

Supplementary Fig. 12. HR-TEM of CPF-Fe/Ni after electrocatalysis testing in 0.5 M H_2SO_4.

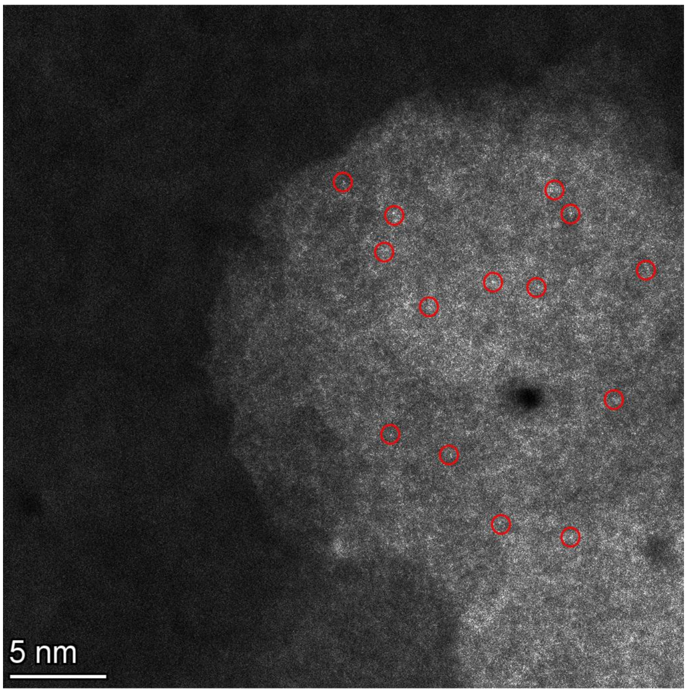

Supplementary Fig. 13. HAADF STEM image of CPF-Fe/Ni after electrocatalysis testing in 0.5 M H_2SO_4.

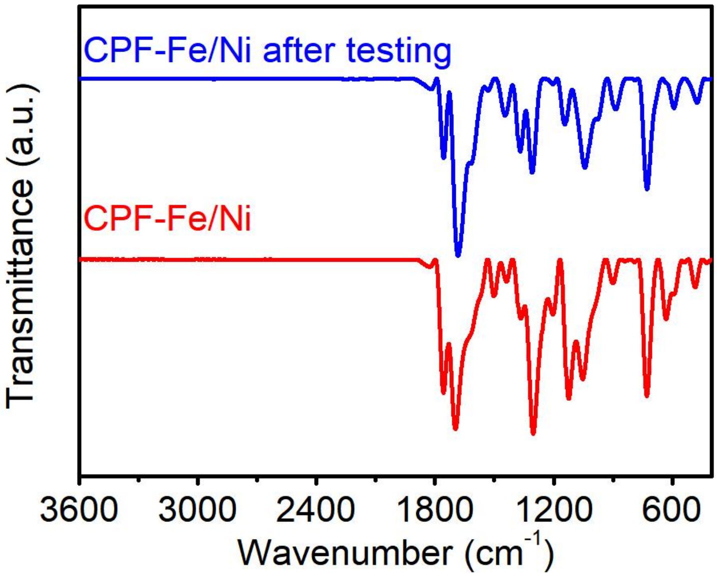

Supplementary Fig. 14. FT-IR of CPF-Fe/Ni after electrocatalysis testing in 0.5 M H_2SO_4.

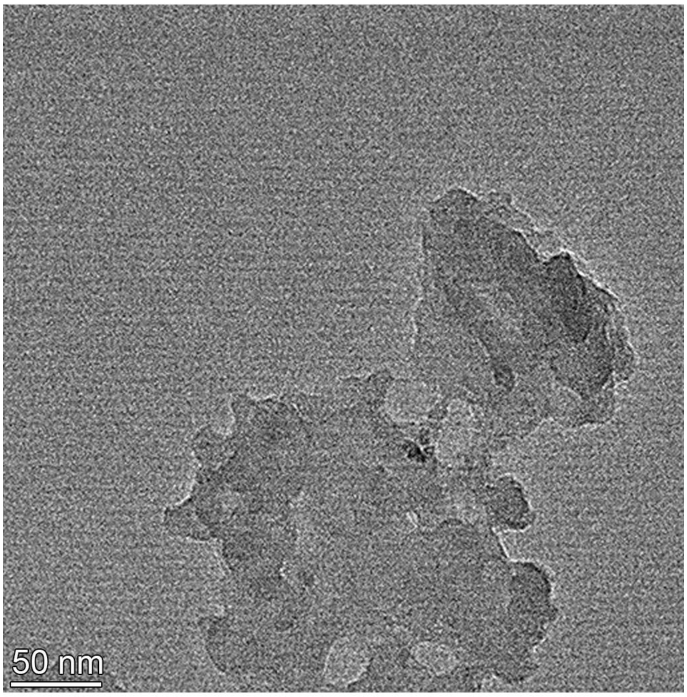

Supplementary Fig. 15. HR-TEM of CPF-Fe/Ni after electrocatalysis testing in 1 M KOH.

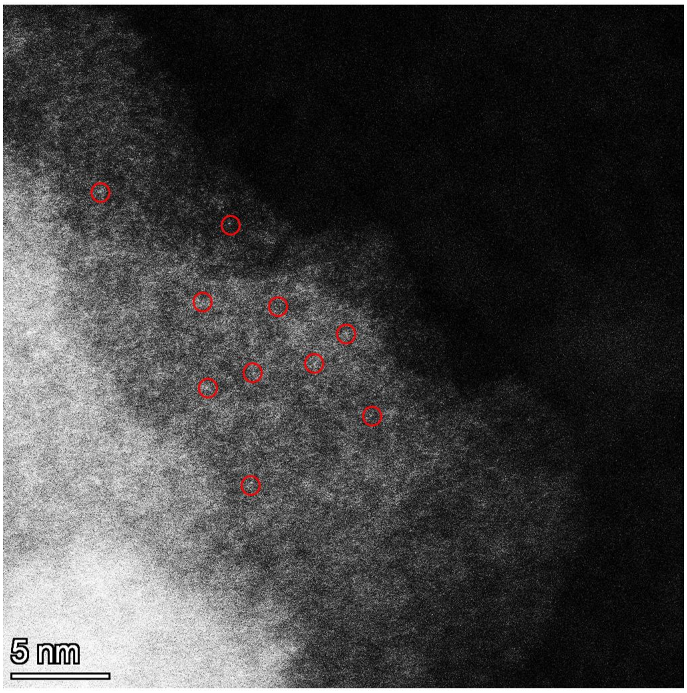

Supplementary Fig. 16. HAADF STEM image of CPF-Fe/Ni after electrocatalysis testing in 1 M KOH.

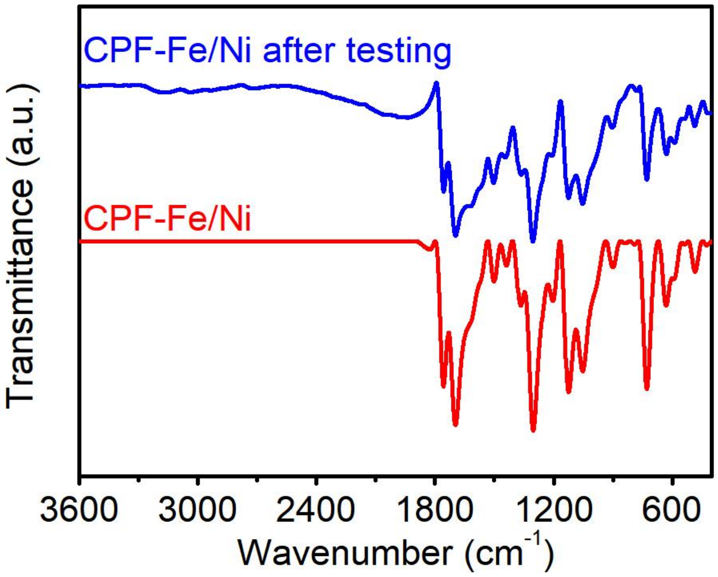

Supplementary Fig. 17. FT-IR of CPF-Fe/Ni after electrocatalysis testing in 1 M KOH.

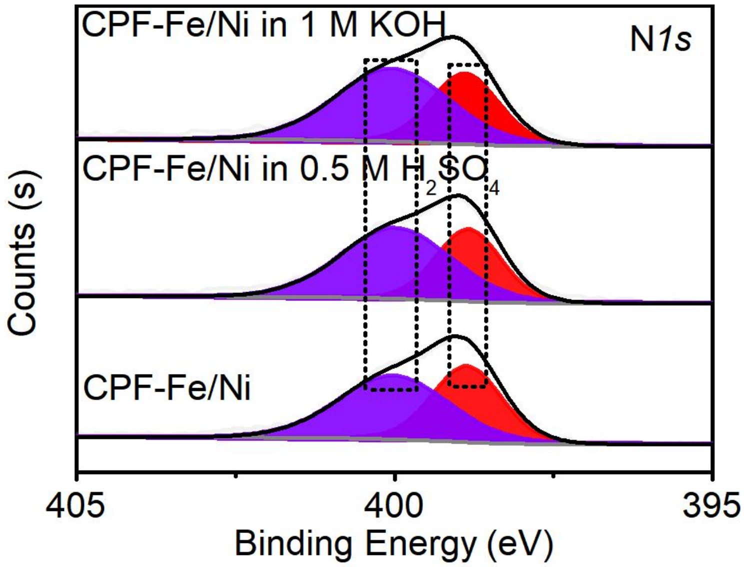

Supplementary Fig. 18. High-resolution N1s spectra of CPF-Fe/Ni after electrocatalysis testing in 0.5 M H_2SO_4 and 1 M KOH, respectively.

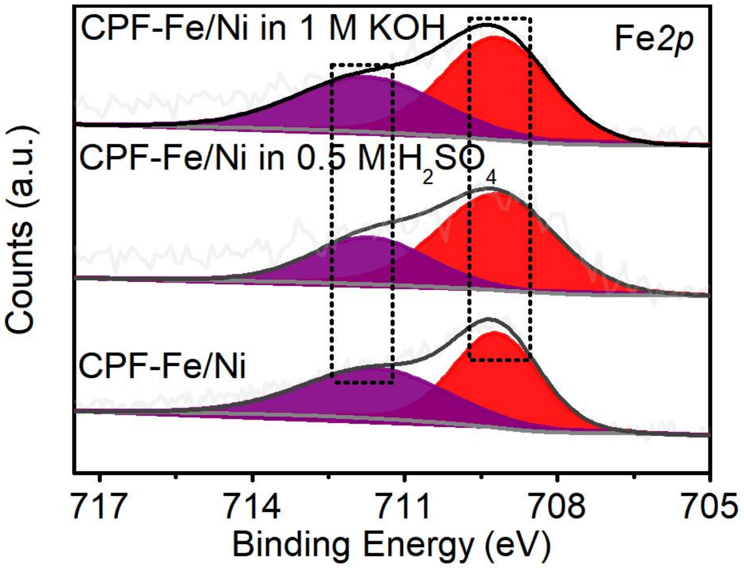

Supplementary Fig. 19. High-resolution Fe2p spectra of CPF-Fe/Ni after electrocatalysis testing in 0.5 M H_2SO_4 and 1 M KOH, respectively.

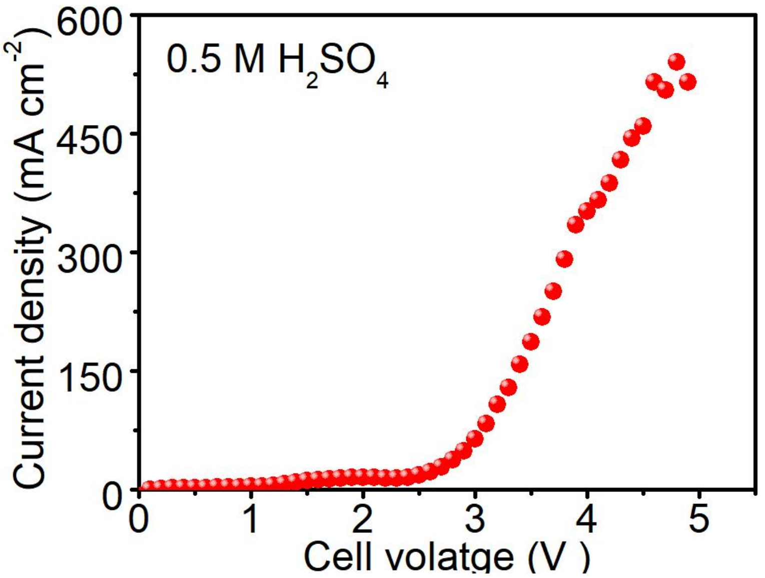

Supplementary Fig. 20. LSV curves of CPF-Fe/Ni coupled water electrolysis cell at a scan rate of 10 mV s^{-1} in 0.5 M H_2SO_4.

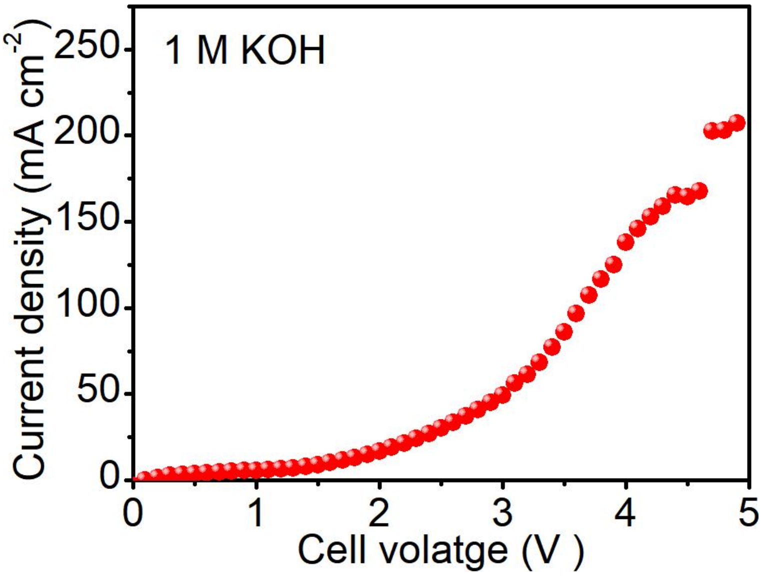

Figure S21. LSV curves of CPF-Fe/Ni coupled water electrolysis cell at a scan rate of 10 mV s^{-1} in 1 M KOH.

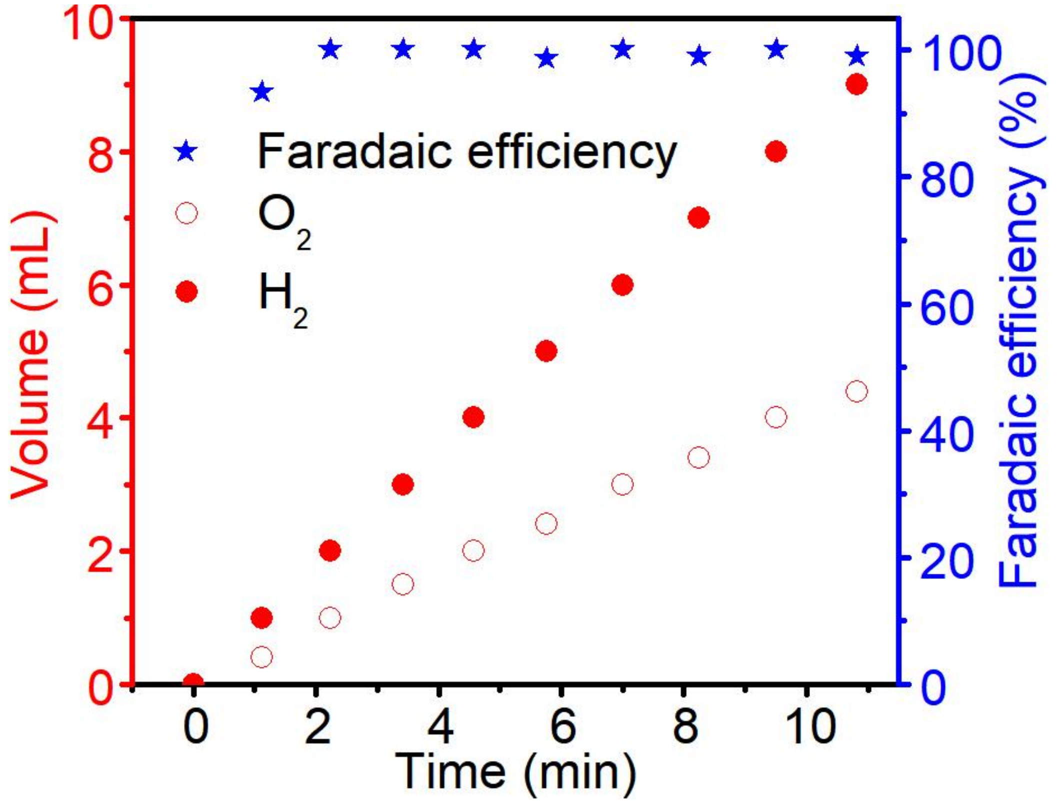

Supplementary Fig. 22. Experimental gas volumes of H_2 and O_2 during water splitting at a current density of 30 mA cm^{-2} for 11 min in 0.5 M H_2SO_4, and the corresponding Faradaic efficiency of CPF-Fe/Ni.

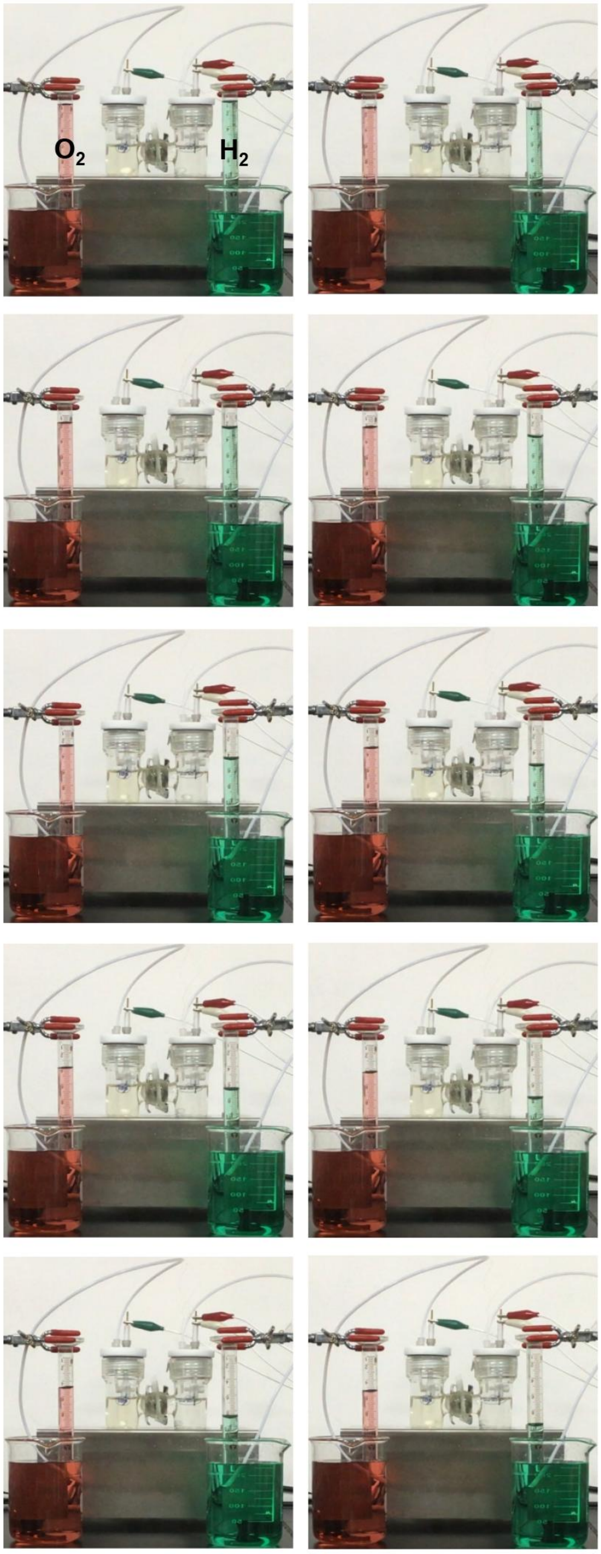

Supplementary Fig. 23. Hydrogen (green) and oxygen (red) generated at 0, 1.12, 2,23, 3.43, 4.58, 5.77, 7, 8.25, 9.5, and 10.83 min in 0.5 M H_2SO_4, respectively.

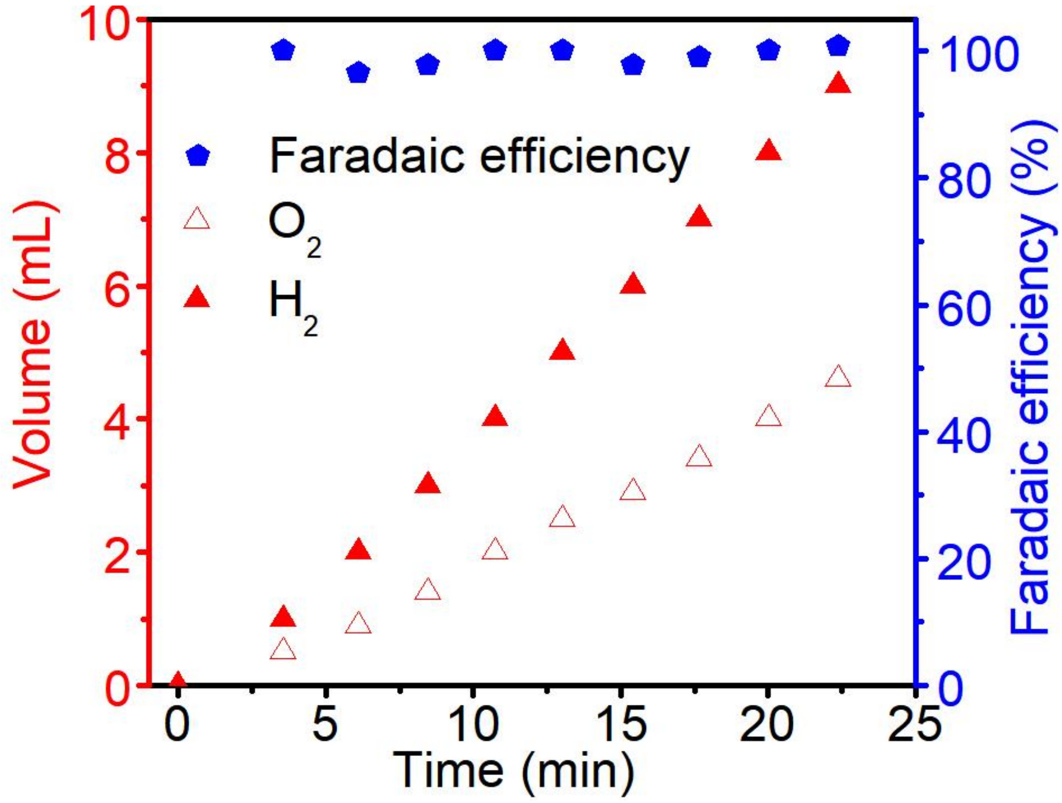

Supplementary Fig. 24. Experimental gas volumes of H_2 and O_2 during water splitting at a current density of 30 mA cm^{-2} for 25 min in 1 M KOH, and the corresponding Faradaic efficiency of CPF-Fe/Ni.

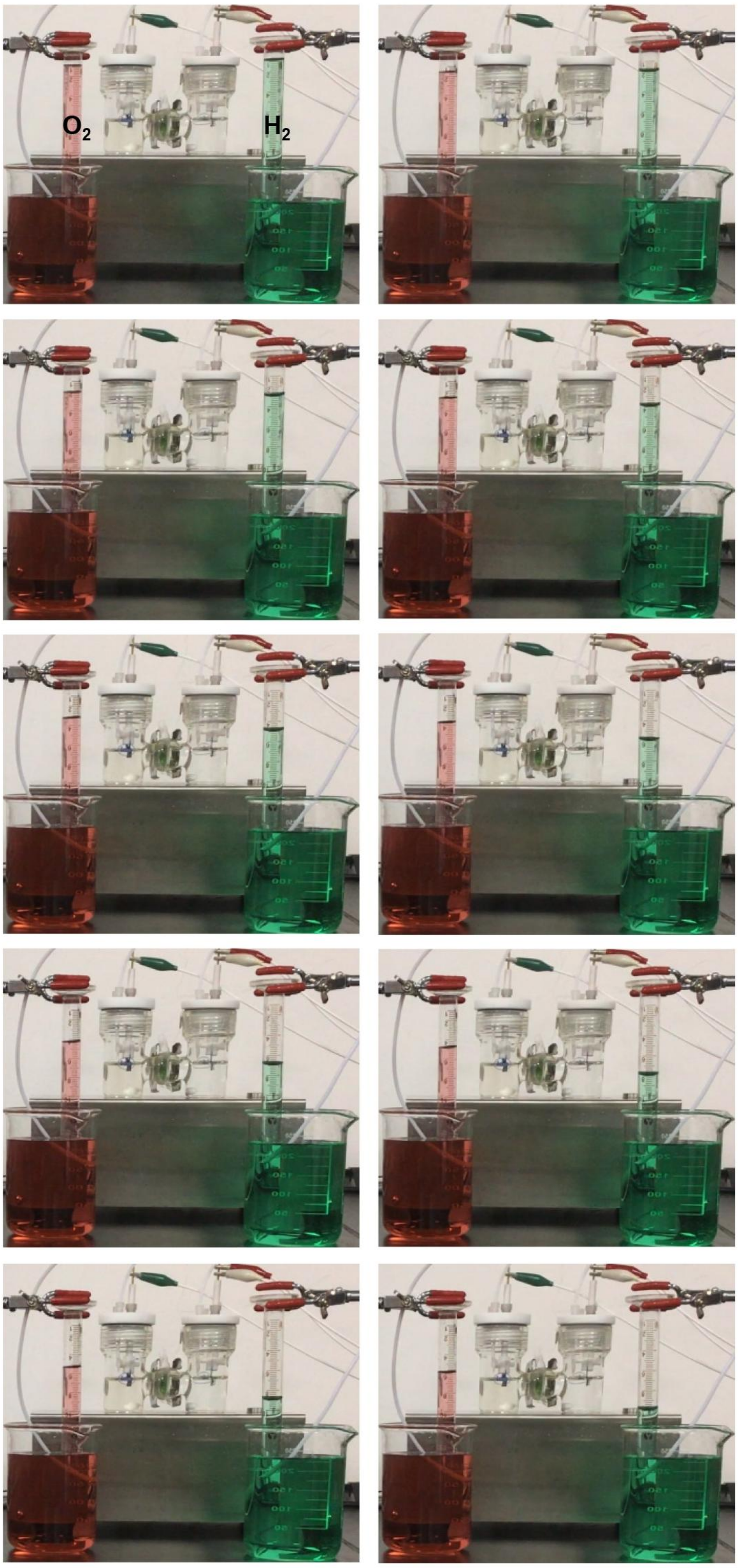

Supplementary Fig. 25. Hydrogen (green) and oxygen (red) generated at 0, 3.55, 6.1, 8.45, 10.76, 13.02, 15.42, 17.67, 20.03, and 22.38 min in 1 M KOH, respectively.

Supplementary Tables

Supplementary Table 1. Comparison of different electrocatalytic activity for different water splitting catalysts under 10 mA cm^{-2}.

	0.5 M H_2SO_4 η HER/OER (mV)	1 M KOH η HER/OER (mV)	0.5 M H_2SO_4 Cell voltage (V)	1 M KOH Cell voltage (V)	Tafel (mVdec^{-1})	TOF (s^{-1})	Faradaic Efficiency (%)	Stability (h)	***Ref***
**CPF-Fe/Ni**	**23/201**	**42/194**	**1.44**	**1.57**	**0.5 M H_2SO_4: 82.6 (HER) 94.1 (OER) 1 M KOH: 169.5 (HER) 102.1 (OER)**	**H_2SO_4: 3.16 (H_2) 1.12 (O_2) KOH: 2.64 (H_2) 1.4 (O_2)**	**~ 100**	**H_2SO_4: 120 KOH: 120**	***Our Work***
Ru/Co–N–C	13/232	23/247	1.49	1.5	0.5 M H_2SO_4: 40.7 (HER) 1 M KOH: 32.4 (HER)	9.2	96	H_2SO_4: 20 KOH: 15	***1***
W-$NiS_{0.5}Se_{0.5}$	--	39/171	--	1.44	1 M KOH: 51 (HER) 41 (OER)	0.21	~ 100	500	***2***
CoNiRu-NT	--	27/255	--	1.47	1 M KOH: 78 (HER) 67 (OER)	0.330	95	48	***3***
FMZP4	--	53/184	--	1.79	1 M KOH: 53.2 (HER) 51.9 (OER)	0.00893	~ 100	80	***4***
Co-Co0.85Se	--	97/265	--	1.47	1 M KOH: 70.7 (HER) 78.0 (OER)	4.4	~ 100	12	***5***
Mn_2P-Mn_2O_3/PNCF	--	98/330	--	1.56	1 M KOH: 46 (HER) 86 (OER)	--	~100	72	***6***
H-CoSx@NiFe LDH/NF	--	95/250	--	1.98	1 M KOH: 90 (HER) 49 (OER)	0.067	97	100	***7***
Co-BTC	--	437/370	--	2.03	1 M KOH: 115.1 (HER) 89.1 (OER)）	1.23	--	5	***8***
BPIr be catalyst	25/290	198/290	1.57	1.54	0.5 M H_2SO_4: 30.9 (HER) 70 (OER) 1 M KOH:	22 (H_2) 4.41 (O_2)	--	H_2SO_4: 2.5 KOH: 2.5	***9***

					91 (HER)				
Ru/RuS_2-2	45/201	--	1.501	--	0.5 M H_2SO_4: 30.9 (HER) 70 (OER) 1 M KOH: 91 (HER) 64 (OER)	0.71 (H_2) 0.61 (O_2)	--	10	***10***
Ru1/D-NiFe LDH	--	10/189	--	1.419	1 M KOH: 29 (HER) 31 (OER)	7.66 (H_2)	~100 (H_2) 99.6 (O_2)	2.5	***11***
D-CoP-HoMSs	--	93/294	--	1.57	1 M KOH: 50 (HER) 67 (OER)	--	--	-	***12***
NiCoPO@NC/P-NF-e	--	73.1/221.4	--	1.5	1 M KOH: 82 (HER) 87.8 (OER)	0.21 (O_2)	98.5 (H_2) 99.4 (O_2)	48	***13***
Ni0.6Fe0.4-MOG	--	159/285	--	1.61	1 M KOH: 38 (HER) 63 (OER)	1.44(H_2) 1.38(O_2)	~ 100	20	***14***
Fe doped MOF CoV@CoO nanoflakes	--	78/220	--	1.53	1 M KOH: 86 (HER) 59 (OER)）	0.45	--	50	***15***
NiMoOx/NiMoS	--	38/186	--	1.46	1 M KOH: 38 (HER) 34 (OER)	1.97	99.6±0.3 (H_2) 97.5±0.4 (O_2)	500	***16***
CoFeO@BP	--	88/256	--	--	1 M KOH: 51 (HER) 42 (OER)	--	~ 100 (H_2) 99.2 (O_2)	24	***17***
CoP-InNC@CNT	--	159/270	--	1.58	1 M KOH: 63 (HER) 85 (OER)）	--	--	15	***18***
Ni@N-HCGHF	--	95/260	--	1.6	1 M KOH: 57 (HER) 63 (OER)	--	--	20	***19***
Co2P /CoNPC	--	208/328	--	1.64	1 M KOH: 83.0 (HER) 72.6 (OER)	--	--	8.3	***20***
D-Ni-MOF NSA	--	101/219	--	1.5	1 M KOH: 50.9 (HER) 48.2 (OER)	1.224		48	***21***
FeNi(BDC)(DMF,F)/NF	--	234/227	--	1.58	1 M KOH: 96.2 (HER) 37.4 (OER)	0.298	~ 100	30	***22***

HOF-Co0.5Fe0.5/NF	--	170/278	--	1.63	1 M KOH: 137 (HER) 59 (OER)	--	99.9	20	***23***

Supplementary Table 2. ICP of CPF-Fe/Ni before and after catalysis.

	Fe (%)	Ni (%)
CPF-Fe/Ni before catalysis	11.90	1.35
CPF-Fe/Ni after catalysis in 0.5 M H_2SO_4	11.16	1.21
CPF-Fe/Ni after catalysis in 1 M KOH	11.32	1.18

Supplementary Note

Structural characterization.

Fourier transform infrared (FT-IR) spectra were recorded on a Bruker ALPHA Ⅱ FT-IR spectrometer.

^{13}C MAS solid-state NMR experiments were performed on AVANCE(3)400WB at a resonance frequency of 150.15 MHz. ^{13}C NMR spectra were recorded with spinning rate of 15kHz with a 4mm probe at room temperature. ^{13}C CPMAS experiments were performed with a delay time of 5s. Scan number: 2048 scans.

The X-ray photoelectron spectroscopy (XPS) measurements and were tested using ESCALAB 250 system (Thermo Electron) with an Al Kα (300 W) X-ray resource (3.82 eV versus absolute vacuum value). All binding energies were calibrated to the C 1s peak (284.6 eV) arising from the adventitious carbon-containing species.

Field-emission scanning electron microscopy (FESEM) measurement was carried out using Zeiss Sigma 500.

The transmission electron microscopy (TEM) and high-resolution transmission electron microscopy (HRTEM) images were obtained in FEI TalosF200S equipment.

Sub-ångström-resolution high-angle annular dark-field scanning transmission electron microscopy (HAADF-STEM) characterization was conducted on a JEOLJEMARM300F STEM/TEM with a guaranteed resolution of 0.08 nm.

Supplementary References

1. Rong C*, et al.* Electronic Structure Engineering of Single-Atom Ru Sites via Co-N4 Sites for Bifunctional pH-Universal Water Splitting. *Adv. Mater.* **34**, 2110103 (2022).
2. Wang Y*, et al.* Highly Active and Durable Single-Atom Tungsten-Doped NiS0.5Se0.5 Nanosheet @ NiS0.5Se0.5 Nanorod Heterostructures for Water Splitting. *Adv. Mater.* **34**, 2107053 (2022).
3. Wang Y*, et al.* Competitive Coordination-Oriented Monodispersed Ruthenium Sites in Conductive MOF/LDH Hetero-Nanotree Catalysts for Efficient Overall Water Splitting in Alkaline Media. *Adv. Mater.* **34**, 2107488 (2022).
4. Huang L, *et al.* In situ phosphating of Zn-doped bimetallic skeletons as a versatile electrocatalyst for water splitting. *Energy Environ. Sci.,* **15**, 2425-2434 (2022).
5. Yu J, et al. Capture and recycling of toxic selenite anions by cobalt-based metal-organic-frameworks for electrocatalytic overall water splitting. *Chem. Eng. J.* **433**, 134553 (2022).
6. Wang X, *et al.* One-pot synthesis of Mn_2P-Mn_2O_3 heterogeneous nanoparticles in a P, N -doped three-dimensional porous carbon framework as a highly efficient bifunctional electrocatalyst for overall water splitting. *Chem. Eng. J.* **428**, 131190 (2022).
7. Lee Y, et al. Metal-Organic Framework-Derived Hollow CoSx Nanoarray Coupled with NiFe Layered Double Hydroxides as Efficient Bifunctional Electrocatalyst for Overall Water Splitting. *Small* **18**, 2200586 (2022).
8. Shreyanka S, *et al.* Multiscale design of 3D metal-organic frameworks (M−BTC, M: Cu, Co, Ni) via PLAL enabling bifunctional electrocatalysts for robust overall water splitting. *Chem. Eng. J.* **446**, 137045 (2022).
9. Mei J, et al. Surface-Dependent Intermediate Adsorption Modulation on Iridium-Modified Black Phosphorus Electrocatalysts for Efficient pH-Universal Water Splitting. *Adv. Mater*. **33**, 2104638 (2021).
10. Zhu J, et al. Regulative Electronic States around Ruthenium/Ruthenium Disulphide Heterointerfaces for Efficient Water Splitting in Acidic Media. *Angew., Chem. Int. Ed*. **60**, 12328-12334 (2021).
11. Zhai P, et al. Engineering single-atomic ruthenium catalytic sites on defective nickel-iron layered double hydroxide for overall water splitting. *Nat. Commun*. **12**, 4587 (2021).
12. Hou P, et al. Delicate Control on the Shell Structure of Hollow Spheres Enables Tunable Mass Transport in Water Splitting. *Angew., Chem. Int. Ed*. **60**, 6926-6931 (2021).
13. Gao H, et al. Nickel-cobalt phosphate nanoparticles wrapped in nitrogen-doped carbon loading on partially phosphatized foamed nickel as efficient electrocatalyst for water splitting. *Chem. Eng. J.* **426**, 130854 (2021).
14. Zhang X, Zhao H, Li C, Li S, Liu K, Wang L. Facile coordination driven synthesis of metal-organic gels toward efficiently electrocatalytic overall water splitting. Appl. Catal. B: Environ. 299, 120641 (2021).
15. Muthurasu A, *et al.* Construction of iron doped cobalt- vanadate- cobalt oxide with metal-organic framework oriented nanoflakes for portable rechargeable zinc-air batteries powered total water splitting. *Chem. Eng. J.* **88**, 106238 (2021).

16. Zhai P, et al. Engineering active sites on hierarchical transition bimetal oxides/sulfides heterostructure array enabling robust overall water splitting. *Nat. Commun*. **11**, 5462 (2020).
17. Li X, et al. Adaptive Bifunctional Electrocatalyst of Amorphous CoFe Oxide@2D Black Phosphorus for Overall Water Splitting. *Angew., Chem. Int. Ed*. **59**, 21106-21113 (2020).
18. Chai L, et al. Stringing Bimetallic Metal-Organic Framework-Derived Cobalt Phosphide Composite for High-Efficiency Overall Water Splitting. *Adv. Sci*. **7**, 1903195 (2020).
19. Yan L, et al. A Freestanding 3D Heterostructure Film Stitched by MOF-Derived Carbon Nanotube Microsphere Superstructure and Reduced Graphene Oxide Sheets: A Superior Multifunctional Electrode for Overall Water Splitting and Zn-Air Batteries. *Adv. Mater*. **32**, 2003313 (2020).
20. Liu H, et al. Metal-Organic-Framework-Derived Co2 P Nanoparticle/Multi-Doped Porous Carbon as a Trifunctional Electrocatalyst. *Adv. Mater*. **32**, 2003649 (2020).
21. Zhou J, *et al.* Alkali-Etched Ni(II)-Based Metal-Organic Framework Nanosheet Arrays for Electrocatalytic Overall Water Splitting. *Small* **16**, 1906564 (2020).
22. Lin H-W, Senthil Raja D, Chuah X-F, Hsieh C-T, Chen Y-A, Lu S-Y. Bi-metallic MOFs possessing hierarchical synergistic effects as high performance electrocatalysts for overall water splitting at high current densities. *Appl. Catal. B: Environ*. **258**, 118023 (2019).
23. Liu FQ, et al. Constructing bimetal-complex based hydrogen-bonded framework for highly efficient electrocatalytic water splitting. *Appl. Catal. B: Environ*. **258**, 117973 (2019).
